# Discovery of a single male *Aedes aegypti* (L.) in Merseyside, England

**DOI:** 10.1186/s13071-017-2251-0

**Published:** 2017-06-24

**Authors:** Thom Dallimore, Tony Hunter, Jolyon M. Medlock, Alexander G.C. Vaux, Ralph E. Harbach, Clare Strode

**Affiliations:** 1 Department of Biology, Edge Hill University, St. Helens Road, Ormskirk, Lancashire L39 4QP UK; 2 Zoology Department, World Museum Liverpool, William Brown Street, Liverpool, L3 8EN UK; 3 Medical Entomology & Zoonoses Ecology group, Emergency Response Department, Public Health England, Porton Down, Salisbury, SP4 0JG UK; 4 Department of Life Sciences, Natural History Museum, Cromwell Road, London, SW7 5BD UK

**Keywords:** *Aedes aegypti*, Invasive, Mosquito, Surveillance, England, UK

## Abstract

**Background:**

The mosquito *Aedes aegypti* (L.) is found in tropical and sub-tropical regions where it is the major vector of dengue fever, yellow fever, chikungunya and more recently Zika virus. Given its importance as a vector of arboviruses and its propensity to be transported to new regions, the European Centre for Disease Prevention and Control (ECDC) has placed *Ae. aegypti* on a list of potentially invasive mosquito species. It was previously reported in the United Kingdom (UK) in 1865 and 1919 but did not establish on either occasion. It is now beginning to reappear in European countries and has been recorded in the Netherlands (not established) and Madeira (Portugal), as well as southern Russia, Georgia and Turkey.

**Results:**

During summer 2014, a single male *Ae. aegypti* was captured during mosquito collections in north-western England using a sweep net. Morphological identification complimented by sequencing of the ITS2 rDNA, and *cox*1 mtDNA regions, confirmed the species. Following confirmation, a programme of targeted surveillance was implemented around the collection site by first identifying potential larval habitats in greenhouses, a cemetery, a farm and industrial units. Despite intensive surveillance around the location, no other *Ae. aegypti* specimens were collected using a combination of sweep netting, larval dipping, mosquito magnets, BG sentinel traps and ovitraps. All species collected were native to the UK.

**Conclusion:**

The finding of the single male *Ae. aegypti*, while significant, presents no apparent disease risk to public health, and the follow-up survey suggests that there was no established population. However, this report does highlight the need for vigilance and robust surveillance, and the requirement for procedures to be in place to investigate such findings.

## Background

The mosquito *Aedes* (*Stegomyia*) *aegypti* (L.) is found in tropical and subtropical regions where it is the vector of arboviruses, such as dengue, chikungunya and yellow fever. This species is also a vector of Zika virus and is currently responsible for widespread cases throughout the Americas [1]. The immature stages of the ancestral form *Ae. aegypti formosus* develop in natural containers (e.g. tree holes, bamboo internodes and leaf axils) but the internationally occurring form *Ae. aegypti aegypti* has adapted its habitat preferences to exploit human-made containers such as water storage tanks, discarded tyres and jars, and water-filled pots. Consequently, it is found near human dwellings making it a particularly effective vector of human diseases. This adaptation to artificial containers, coupled with the ability of *Aedes* eggs to withstand prolonged periods of desiccation, has led to its invasion of new territories globally.

Increasing urbanisation and globalisation, including international trade, have been implicated in the passive dispersal of invasive mosquito species (IMS) such as *Ae. aegypti* and to a greater extent *Ae. albopictus* (Skuse). The international trade in used tyres, lucky bamboo and wet-footed plants, in particular, have all been implicated in the movement of IMS between countries and continents. *Ae. aegypti* introductions into the Netherlands, for instance, was via the importation of used tyres from Miami, Florida [2].

**Fig. 1:**
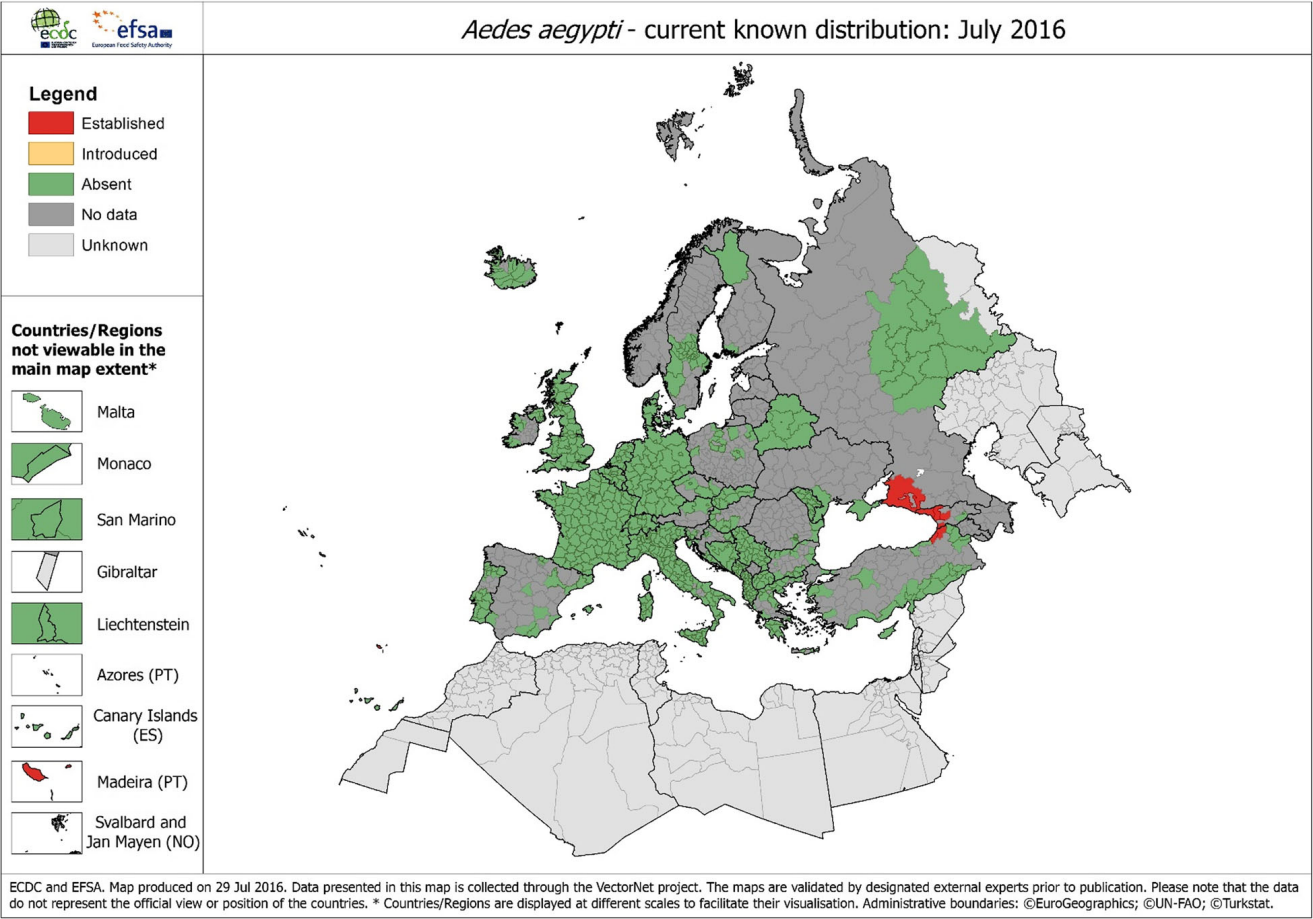
Current known European distribution of *Aedes aegypti*, July 2016 (Source: ECDC-EFSA/VECTORNET)

The European Centre for Disease Prevention and Control (ECDC) considers IMS a serious public health threat to Europe and has produced guidelines for the surveillance of such species [3]. While *Ae. albopictus* remains the most prolific IMS in Europe, having greatly expanded its range across 28 countries, the geographical extent of *Ae. aegypti* in Europe is much more limited. Historically, *Ae. aegypti* occurred widely throughout the Mediterranean but largely died out in the post-WW2 period [4]. However, it has begun to re-colonise parts of southern and south-eastern Europe with populations found in Madeira (Portugal) and the Black Sea coast of Russia, Georgia and more recently Turkey [5, 6] (Fig. 1). Unlike *Ae. albopictus*, which has adapted to cooler climates by entering winter diapause, *Ae. aegypti* has not become established in northern Europe. It has never been recorded as established further north than 44°30′N latitude and its distribution is limited to areas with a January isotherm of 10 °C and mean annual temperatures of 15 °C, making northern Europe including the UK, inimical for their survival [4]. The species was responsible for an outbreak of yellow fever in Swansea, Wales, in 1865 where *Ae. aegypti*, introduced via shipping, were reported to transmit the virus from infected sailors to the local population. The mosquitoes were not recorded as having survived the winter [7].

Within the UK, IMS surveillance includes both passive and active operations [8]. Passive surveillance has involved the collection of existing and historical data on mosquito distributions in the UK, as well as an identification service for mosquitoes collected by entomologists, academics, environmental health officers and members of the public (e.g. the Mosquito Recording Scheme and Mosquito Watch) [8–10]. Active surveillance includes deploying traps and performing larval sampling at strategic sites such as seaports and airports, used tyre import yards, and motorway service stations close to southern ferry ports and the Eurotunnel. Prior to this finding, there had been no reports of IMS via either passive or active surveillance.

## Methods

### Mosquito collections

**Fig. 2:**
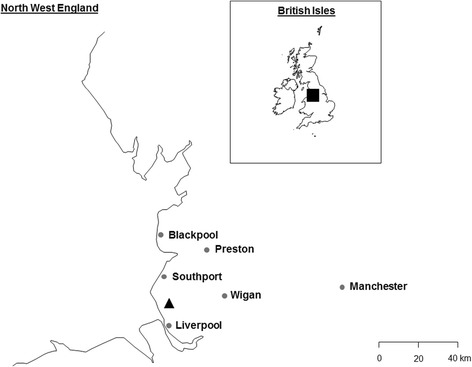
Location of the collection site of the *Ae*. *aegypti* specimen. Triangle indicates point of discovery

A single male mosquito was collected on 13.07.2014 during sweep netting of ferns and other low vegetation, in and around a young mixed broadleaf plantation (<10 years old) 6 km to the north of Liverpool, England (53°30′42.13″N, 2°59′01.74″W) (Fig. 2). The location was ~100 m from an active arable farm yard, and ~250 m from a recently established wetland nature reserve, of wet grassland, fen, reed bed and open water (77 km^2^) (Fig. 2).

### Morphological identification

Specimen identification was undertaken using the keys of Becker et al. [11] and Schaffner et al. [12], and identification was confirmed at the Natural History Museum (NHM), London, by further examination and genitalia dissection.

### Genetic identification

To further confirm the identification, DNA was extracted from a single leg using a Qiagen DNeasy® Blood and Tissue kit (Manchester, UK) according to the manufacturer’s instructions and amplified by PCR. Target amplification was carried out using Phusion® high-fidelity polymerase (New England Biolabs. Hitchin, UK) on the internal transcribed spacer 2 (ITS2) of ribosomal DNA (rDNA), and the mitochondrial cytochrome oxidase I (*cox*1) region using the following 5′-TGT GAA CTG CAG GAC ACA TG-3′ (ITS2 forward) and 5′-ATG CTT AAA TTT AGG GGG TA-3′ (ITS2 reverse) primers of Walton et al. [13], and 5′-GGT CAA CAA ATC ATA AAG ATA TTG G-3′ (*cox*1 forward) and 5′-TAA TAT GGC AGA TTA GTG CAT TGGA-3′ (*cox*1 reverse). The PCR products were purified using the ThermoFisher Scientific GeneJET Purification Kit(Paisley, UK), and amplification success was confirmed by gel electrophoresis. The products were subsequently sequenced using an Applied Biosystems 3730 DNA Analyser with BigDye v. 3.1 (University of Shuffled, Core Genomic Facility). The sequences were then blasted in GenBank® for sequence similarity matches [14]. DNA sequencing was replicated five times for ITS2 and three times for *cox*1 regions to remove discourse through PCR error.

### Targeted surveillance

As part of the ongoing sampling of mosquitoes, and prior to the confirmation of the *Aedes* specimen, sweep netting, and larval dipping close to the point of discovery (POD) was conducted by Edge Hill University (EHU) and the Liverpool World Museum in August 2015. Additionally, three Mosquito Magnet® Independence traps were deployed within the adjacent wetland nature reserve ~200 m, ~ 450 m and ~1000 m from the POD: the first was positioned adjacent to a vegetated drainage ditch, the second close to a blocked water filled ditch and an open water pond. The third was placed in an area of wet grassland.

Once the specimen was confirmed as *Aedes aegypti* a programme of targeted surveillance was implemented by entomologists at EHU and Public Health England (PHE). Consideration of species dispersal was taken into account as *Ae. aegypti* has been reported to have poor dispersal capabilities, with approximately a 50–363 m mean life-time dispersal distance [15–17]. Merseyside and West Lancashire comprises flat open expanses that are often subject to high winds so normal dispersal distance may be exaggerated in these conditions. As a precaution, a 2 km^2^ search area was designated around the POD to search for established populations. Potential larval habitats within the search area were identified using a combination of local knowledge, on the ground investigations and Google Maps [18]. The strategy was developed utilising the ECDC guidelines [3].

The landscape surrounding the POD is predominately arable farmland and residential housing. Four locations found within this area were considered as potential *Ae. aegypti* habitat: an active farm yard with used tyres (~1 km^2^), a disused garden centre with extensive greenhouses (~0.6 km^2^), a large cemetery with numerous flower vases (~9 km^2^) and an industrial estate (~9 km^2^), with distances from the POD of ~100 m (S), 1300 m (SW), 1600 m (SW) and 2000 m (ESE), respectively. Surveillance was concentrated within these areas using BG-Sentinel (Biogents) adult traps and ovitraps, with larval dipping, also carried out where appropriate (Table 1). The BG-Sentinels were deployed with CO2 and BG-Sweetscent™ lures and run on 12 V car batteries that were re-charged weekly. The traps were activated on all sites from 9–11th September 2015 until the 27th October 2015. A total of 38 ovitraps where deployed concurrently with the BG-Sentinels at three of the target sites (with only the BG-Sentinel deployed at the industrial estate). Traps were checked once per week.

**Table 1:** Types of mosquito traps deployed at potential *Ae*. *aegypti* larval habitats

Method of mosquito surveillance	Surveillance site					
	Forestry plantation	Wetland Nature Reserve	Active farm yard	Disused garden centre	Cemetery	Industrial estate
Sweep net	Yes	No	Yes	No	No	No
Larval dipping	Yes	Yes	Yes	Yes	No	Yes
Mosquito Magnet®	No	Yes	No	No	No	No
BG-Sentinel trap	No	No	Yes	Yes	Yes	Yes
Ovitrap	No	No	Yes	Yes	Yes	No

*Yes* trap deployed, *No* trap not deployed

The working farm was the closest site to the POD with suitable larval habitats including water-filled containers, blood sources and shelter in farm buildings. The industrial estate was selected due to the presence of tyres, which on inspection were newly manufactured and not stored outside for long enough periods of time that would allow water to accumulate, this was, therefore, an unlikely source of introduction. Transportation of horticultural goods has proven to be an active method of IMS movement [19, 20] and the disused garden centre provided potential larval habitat for *Ae. aegypti* with water-filled containers. Several large greenhouses were also present providing shelter and higher temperatures. This could have potentially permitted over-wintering. This site has been demolished since this investigation. Cemeteries have proven to be ideal sites for container inhabitants such as *Ae. aegypti*, with an abundance of water-filled flower vases, sugar source from flowers, blood sources from cemetery workers, visitors, birds and animals, as well as providing shelter around grave stones and surrounding trees and vegetation [21]. A local cemetery was identified as a priority for surveillance, and at the request of the cemetery owners, sampling was limited to methods that were inconspicuous (e.g. ovitraps and BG-Sentinels) to respect the sensitivity of the location. Therefore, larval dipping of vases at grave sites was not undertaken.

### Literature search

A comprehensive search of historical records for *Ae. aegypti* was undertaken to determine if the species had previously established itself in the UK. This included museum records, historical journal articles and grey literature sources. Data from the NBN Gateway [22], Merseyside BioBank [23] and Mosquito Recording Scheme/Mosquito Watch [24] biological recording centres were also searched.

## Results

### Morphological identification

**Fig. 3:**
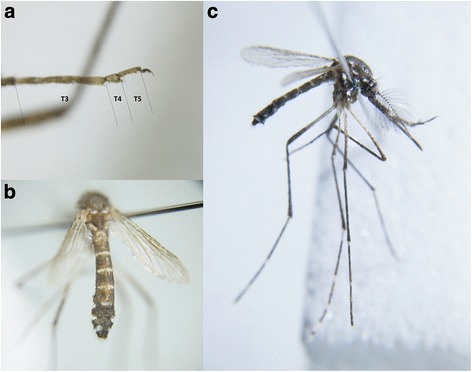
**a** Foreleg tarsomeres 3–5. **b** Dorsal view of the abdomen and wings. **c** Lateral view

On discovery, the specimen was in a reasonably good condition except missing scutal scaling, a foreleg and tarsomere five from one of the hindlegs. The validity of the identification using the key by Schaffner et al. [12] was questioned, as the length of tarsomere 4 was observably shorter than tarsomere 5. This feature is used as a generic characteristic of *Orthopodomyia* and resulted in an initial misidentification. Defacement of scales on the scutum also made clear determination difficult as the diagnostic lateral lyre-shaped white lines were not clearly visible [3]. As a result, additional confirmation was sought from the NHM. Further careful examination and dissection of the genitalia were required to make and confirm identification, respectively, of the specimen as *Ae. aegypti*. The specimen (Fig. 3) is deposited in the NHM collection (Specimen barcode no. 010630631).

### Genetic identification

Blasted *cox*1 and ITS2 regions were shown to match a number of *Ae. aegypti* sequences in the GenBank database. The closest matches to the *cox*1 was 100% identity to KY022527 and for ITS2 100% identity to KF471584. Sequence data from this study was deposited within the GenBank database (accession numbers; BM9ITS2, MF043260 and BM9COI, MF043259).

### Targeted surveillance

A total of 366 mosquitoes (161 adults, two pupae and 203 larvae) were collected across all the surveillance sites, with six species identified (Table 2). Species recorded in the order of greatest abundance were *Culex pipiens* (L.) (*s.l*.), *Anopheles claviger* (Meigen), *Culiseta annulata* (Schrank), *Cs. morsitans* (Theobald), *Cx. torrentium* (Martini) and *Ae. caspius* (Pallas). No specimens of *Ae. aegypti* were found.

**Table 2:** Mosquito species found at the various surveillance sites based on collection method

Method of mosquito surveillance	Surveillance site					
	Forestry plantation	Wetland Nature Reserve	Active farm yard	Disused garden centre	Cemetery	Industrial estate
Sweep net	*An. claviger*, *Cs. annulata*, *Cs. morsitans*, *Cx. pipiens* (*s*.*l*.)	na	*Cx. pipiens* (*s*.*l*.)	na	na	na
Larval dipping	None	*An. claviger*	*An. claviger*, *Cs. annulata*, *Cx. pipiens* (*s*.*l*.), *Cx. torrentium*	*Cx. pipiens* (*s*.*l*.), *Cx. torrentium*	na	None
Mosquito magnet	na	*An. claviger*, *Cs. annulata*, *Cs. morsitans*, *Ae. caspius*	na	na	na	na
BG sentinel trap	na	na	None	*Cx. pipiens* (*s*.*l*.)	None	None
Ovitrap	na	na	*An. claviger*	None	None	na

*Abbreviation*: *na* not applicable, as trap was not deployed

Searching by dipping and sweep netting was by far the most productive method of sampling, followed by the Mosquito Magnets®, ovitraps and the BG-Sentinels, respectively. Both the BG-Sentinels and the ovitraps captured very few specimens. These traps are designed for IMS and do not regularly capture *Cx. pipiens* (*s*.*l*.) in the UK.

Most specimens were found at the active farm yard, and the wetland nature reserve, with no specimens recorded at the cemetery or the industrial estate. However, dipping and netting were limited in both locations due to restrictions on site activity, therefore trapping methods were limited to BG-Sentinels and ovitraps. As the primary aim of the surveillance was to find *Ae. aegypti*, which is known to be effectively surveyed by both methods, the lack of specimens found is indicative of species absence [25, 26].

## Discussion

Given that only a single male specimen was found in 2014 and no other individuals were collected during surveys in 2015, it can be assumed that *Ae. aegypti* was not locally established. Furthermore, any population would be unlikely to reach its biotic potential. There are several well-recorded factors that can affect the fecundity of *Ae. aegypti*, namely food availability [27, 28], suitability of the physical environment [29], humidity [27] and particularly temperature [30–34]. However, attempts to determine the survival ability of *Ae. aegypti* at different temperatures has been heavily weighted towards laboratory-based experiments rather than studies in the field [35]. Additionally, little research has been done to establish the adaptability of *Ae. aegypti* at the extremes of its temperature range. Despite this gap in the available literature, current estimates for *Ae. aegypti* survival range from 10 to 35 °C for adults [35] and 10–30 °C for larvae [31], although the successful development of larvae, and the metabolising of food, is difficult at the extremes.

Northerly latitudes have previously been considered unsuitable for the establishment of *Ae. aegypti*. Our current knowledge of the life history of this species suggests that it is unable to survive winters at these extremes. The temperature thresholds for the persistence of *Ae. aegypti* populations are thought to be the January isotherm of 10 °C or the annual mean temperature of 15 °C (see Schaffner & Mathis [4]). To put this in context, January isotherms for Scotland are 4–5 °C, and in England mostly 5–6 °C with 7 °C in SW Cornwall. According to the UK Met Office (officially the Meteorological Office until 2000) in January 2016 mean temperatures were 5.4 °C in Wales, 5.2 °C in England, 5.0 °C in Northern Ireland and 3.0 °C in Scotland. Records for January 2015 were colder. In some years (2001–2016) some parts of London and the south coast experienced mean January isotherms above 6 ^o^C, with > 8 °C reported in a few localities. Annual mean temperatures across the UK (1981–2010) vary between 4 and 11 °C, with > 11 °C in parts of London and the south coast of England. It is unlikely, therefore, that *Ae*. *aegypti* would establish in the UK [36]. This is supported by the discovery and subsequent monitoring of *Ae*. *aegypti* in the Netherlands [37]. However, to accurately predict the extension of its range, *Ae*. *aegypti* behavioural studies are needed to determine if urban refugia, such as heated houses, are a potential resource for assisted overwintering.

For IMS to establish in a new territory and overwinter, their population size must be large enough not to suffer from a lack of genetic variation [20]. Regions in southern Germany, for example, have suffered repeated re-introductions of *Ae. albopictus* via ground transport [38]. The UK benefits from being a small island compared to the large landmass of continental Europe, so re-introductions may not be as common.

In this instance, we were unable to determine the point of entry for the specimen. The working farm was the closest site to the POD that contained suitable breeding habitats including water-filled containers, blood sources and shelter in farm buildings. From a site survey conducted at the time of the surveillance, there were no obviously introduced/planted material in the mixed broad-leaf plantation which would otherwise be a risk for the introduction of IMS. The site was planted 11 years ago with native species with minimal subsequent intervention. The industrial estate was selected due to the presence of tyres which on inspection during active surveillance were in fact newly manufactured and not stored outside for periods of time long enough that would allow water to accumulate. We still believed it was prudent to continue with monitoring at this site.

Transport of horticultural goods has been demonstrated as a method of IMS movement, as such the garden centre had been disused for several years and presented an ideal breeding site for *Ae. aegypti*, as there was plenty of water filled containers and the greenhouses, presented ideal shelter and warmth for adult mosquitoes. Surveillance time at the garden centre was limited due to its scheduled demolition for a building development. Despite the time restriction, no additional IMS were found.

The initial identification by EHU using the morphological keys of Cranston et al. [39], for mosquitoes in the UK, and Schaffner et al. [12], for mosquitoes in Europe, was not straight forward. It proved that morphological features alone could make identification difficult if the specimen is missing key features and, particularly in this case if it belongs to a non-endemic species not included in regional specific keys. This situation has highlighted the need for supplementing morphological identification with genetic methods to circumvent these issues, which are important to IMS surveillance projects.

The recent introduction of *Ae*. *aegypti* into the Netherlands [40] and the rapid response to eliminate this population, along with the specimen reported here, highlights the continued need for passive and active surveillance methods for mosquito reporting, as highlighted by Vaux and Medlock [8]. We encourage individuals collecting mosquitoes in the UK, either through entomological work and environmental health investigations of nuisance reporting, to submit specimens to entomologists at PHE, NHM or EHU for identification. This record of *Ae*. *aegypti* remains enigmatic and based upon the evidence presents no public health concern.

## Conclusions

The discovery of a single *Ae*. *aegypti* male mosquito in the North-West region of the UK leads to targeted surveillance of the local area. As no other specimens were found, there is no risk to public health. Despite this, this study demonstrates the need to for surveillance and vigilance in countries believed to be climatically unsuitable for *Ae*. *aegypti* and other invasive mosquito species that pose a health risk. It is equally important that procedures are in plan to deal with situations such as the one encountered in this study.
